# Pre-COVID-19 international travel and admission to hospital when back home: travel behavior, carriage of highly resistant microorganisms, and risk perception of patients admitted to a large tertiary care hospital

**DOI:** 10.1186/s13756-022-01106-x

**Published:** 2022-06-02

**Authors:** Anne F. Voor in ’t holt, Adriënne S. van der Schoor, Kees Mourik, Nikolaos Strepis, Corné H. W. Klaassen, Margreet C. Vos, Juliëtte A. Severin

**Affiliations:** Department of Medical Microbiology and Infectious Diseases, Erasmus MC University Medical Center, Rotterdam, The Netherlands

**Keywords:** Travel, Travel-related illness, Enterobacteriaceae, Enterobacterales, Drug resistance, Risk factors, Surveys and questionnaires, Perception, Bacteria, Behavior

## Abstract

**Background:**

When people who recently travelled abroad are admitted to a hospital back home, there is a risk of introducing highly resistant microorganisms (HRMO) into the hospital. To minimize this risk, a feasible infection prevention strategy should be developed. In this study, we investigated patients’ travel history and behavior during travel and analyzed whether this was correlated to HRMO carriage at admission.

**Methods:**

From May 2018 until August 2019, adult patients admitted to a large tertiary care center in the Netherlands were asked upon hospital admission to participate in the study. Included patients received a questionnaire about risk perception, travel history in the last year, and behavior during travel, and were screened for HRMO carriage at admission using a perianal swab.

**Results:**

Six hundred and eight questionnaires were handed out, of which 247 were returned (40.6%). One hundred and thirty (52.6%) patients did not travel abroad in the last year, of whom eight (6.2%) were HRMO carrier at admission. One hundred seventeen (47.4%) patients travelled in the preceding year, of whom seven patients (6.0%) were HRMO carrier at admission. Thirty patients (12%) travelled outside of Europe; in this group HRMO prevalence was 13.3% (4 out of 30). The majority of patients (71.3%) were aware that international travel could lead to carriage of HRMO, and an even larger majority (89.5%) would support a screening strategy upon hospital admission in case of a travel history, to minimize the risk of introducing HRMO.

**Conclusions:**

We identified that half of admitted patients to a large tertiary care hospital travelled abroad in the last year, with only a small percentage outside Europe. We discuss several screening strategies and propose a strategy of screening and preemptive isolation of patients who travelled to Asia or Africa in the 2 months before their hospital admission; a strategy that patients would support.

**Supplementary Information:**

The online version contains supplementary material available at 10.1186/s13756-022-01106-x.

## Background

Before the start of the SARS-CoV-2 pandemic, international tourism was on the rise worldwide. Tourism increased from 25 million tourist arrivals in 1950 to over 1.4 billion international tourist arrivals in 2019 [1]. Although the number of tourist arrivals has fallen to around 380 million in 2020, it is expected that it will return to the 2019 levels within 2.5 to 4 years [2]. These international travelers do pick up microorganisms that they are exposed to during travel, among which antibiotic-resistant bacteria, and bring these microorganisms back home [3].

In recent years, it has been increasingly recognized that highly resistant microorganisms (HRMO) are a threat to human health, hampering antibiotic therapy, and increasing morbidity and mortality, especially in patients admitted to hospitals. Important risk factors for acquiring HRMO while travelling are exposure to healthcare abroad, experiencing travelers’ diarrhea, and/or antibiotic use during travel. Travel to certain destinations is also a risk factor, specifically to Southern Asia; which is known as a region with high HRMO prevalence [4]. A recent Dutch study amongst healthy travelers showed that 34.3% of included persons acquired extended-spectrum beta-lactamase (ESBL)-producing bacteria during travel, with an astonishing 75.1% in travelers travelling to Southern Asia [3]. Other known risk factors include for example ice cream consumption, and consuming meals at street food stalls [4]. Protective factors, although not well established, have also been identified; such as handwashing before meals, and having a vegetarian diet [3–5].

It is assumed that there is an increased risk of introducing HRMO into the hospital when people from countries with a low prevalence of HRMO are admitted to a hospital, after they have returned from travelling to countries with a high prevalence of HRMO. To contain this risk, a strategy that includes questions at admission about travel history, preemptive isolation, and screening for HRMO could be developed. However, it is unknown how many patients travel and to which destinations, and if they indeed carry HRMO at admission. Therefore, the primary aim of this study was to investigate the travel behavior of patients admitted to a large tertiary care hospital in a country with low prevalence of HRMO, and to correlate travel behavior to HRMO carriage of patients at admission. The secondary aim was to gain insight in the travel-related risk perception of patients, and about their opinion regarding measures hospitals can implement to prevent HRMO transmission due to undetected carriers. This knowledge can then be used in the future to develop policies or guidelines. Furthermore, we aimed to determine by whole genome sequencing (WGS) the sequence types and antimicrobial resistance genes in HRMO identified from travelling and non-travelling patients.

## Methods

### Study design

The Erasmus MC University Medical Center (Erasmus MC) Rotterdam, the Netherlands, is a tertiary care, university hospital, with all medical specialties available. In 2018, the Erasmus MC relocated to a newly constructed hospital building (*i.e.* for adult patients only), which opened for admissions at May 18, 2018. The new hospital consisted of 522 single-occupancy rooms with private bathrooms.

This prospective cohort study included patients admitted from May 18, 2018 until September 1, 2019. Adult patients admitted to departments cardiology, gastroenterology and hepatology, general surgery, hematology, internal medicine, nephrology, neurology, neurosurgery, orthopedics, or plastic surgery with an expected stay of more than 48 h were asked to participate at admission. Patients with multiple hospitalizations during the study period were allowed to participate more than once. Participating patients received a questionnaire with accompanying return envelope, and a perianal swab (flocked swab [ESwab Copan Italia, Brescia, Italy] was obtained within 24 h of admission and transported in its accompanying 1 mL Amies medium). Samples were taken by trained members of the research team, or patients could self-sample with instructions from the members of the research team.

### Questionnaire

A questionnaire and a patient information form were designed in Dutch (see Additional file 1). The questionnaire was pilot tested on three persons and adjusted accordingly. The questionnaire included questions about risk perception (*i.e.* awareness and feelings about international travel and risk of acquiring HRMO), contact with domestic and farm animals, antibiotic use < 1 year, antacid use < 1 year, travel history < 1 year of persons living in the same household, and travel history of the patient < 1 year. If patients did travel, questions were asked about behavior during travel (*e.g.* pastry and ice cream consumption), use of malaria prophylaxis, experiencing travelers’ diarrhea and/or vomiting, hospitalization, antibiotic use, and antacid use during travel.

### Microbiological methods

Samples collected from May 18, 2018, until January 19, 2019, were stored in a − 80 °C freezer before being processed. To prevent freezing/defrosting damage, 0.2 mL 99% glycerol was added to the samples before freezing. Samples taken after January 19, 2019 were processed directly. All samples, regardless of being frozen, were processed using the same procedure. Samples were screened for highly resistant *Pseudomonas aeruginosa*, -*Acinetobacter baumannii*, -*Enterococcus faecium,* and -Enterobacterales. First, 250µL was placed in an Enterococcosel Broth (BD diagnostics, Sparks, USA) with amoxicillin 8 mg/L and incubated overnight at 35 °C. From this broth, a Vancomycin Screen Agar (VSA, BD diagnostics, Sparks, USA) plate was inoculated and incubated twice overnight at 35 °C. Second, 250 µL was placed in a tryptic soy broth with vancomycin (50 mg/L) and incubated overnight at 35 °C. From the vancomycin broth, a ChromID Carba Smart plate (bioMérieux, Marcy l’Etoile, France) was inoculated on both sides and incubated overnight twice at 35 °C. Additionally, from the vancomycin broth, a BrillianceTM ESBL Agar (Oxoid, Basingstoke, UK) was inoculated and incubated twice overnight at 35 °C. For all plates, colonies were identified using MALDI-TOF MS (Bruker Daltonik, Bremen, Germany). In case of *P. aeruginosa*, isolates were tested for the presence of *bla*_OXA-48_, *bla*_KPC_, *bla*_IMP_, *bla*_VIM_, *bla*_NDM_ genes, using PCR, with use of established procedures. When negative, a Carbapenem Inactivation Method (CIM) test was performed [6]. For *A. baumannii* isolates and for ESBL suspected colonies, antibiotic susceptibility was tested using VITEK-2 (bioMérieux, Marcy l’Etoile, France). When *A. baumannii* isolates and ESBL suspected isolates were also suspected for carbapenemase production, a CIM test was performed. For isolates identified as *E. faecium*, a *vanA/vanB* PCR was performed (using established procedures, unpublished).

### Genome sequencing and analysis

To assess sequence types and presence of antimicrobial resistance genes, WGS was performed for all detected HRMO.

DNA was extracted using MagNA pure 96 (Roche Applied Science, Mannheim, Germany). DNA sequencing was performed by Novogene (Beijing, China) using Illumina chemistry creating 150 bp paired end reads. Assemblies were created using Unicycler v0.4 with default parameters [7]. Antimicrobial resistance genes were detected with RGI v5.1.0 using CARD database v3.0.5. Assembled genomes from *Escherichia coli* and *Klebsiella pneumoniae* were processed using the wgMLST scheme available in SeqSphere v5.1.0 (Ridom, Munster, Germany) (https://www.ridom.de/seqsphere/). Clustering trees and heatmaps were generated in R.

### Statistical analysis

Data was presented as percentages, medians or means. In case of multiple visited regions, the region where the patient stayed the longest was used for analysis. The variable age was determined using date of birth and the date of filling out the questionnaire. Differences between groups were identified using the Chi-square statistic, T-test or if not normally distributed the independent-samples Mann–Whitney U test,using SPSS version 21 (IBM Corp., Armonk, New York, USA). *P*-values < 0.05 were considered statistically significant.

### Ethics statement

Written approval to conduct this study was received from the Medical Ethical Research Committee of the Erasmus MC (MEC-2017-1011). This study was not subjected to the Medical Research Involving Human Subjects Act. All patients participating in this study provided written informed consent. This study is registered in the Dutch National Trial Register (trial NL8406).

## Results

### Patient characteristics

**Fig. 1:**
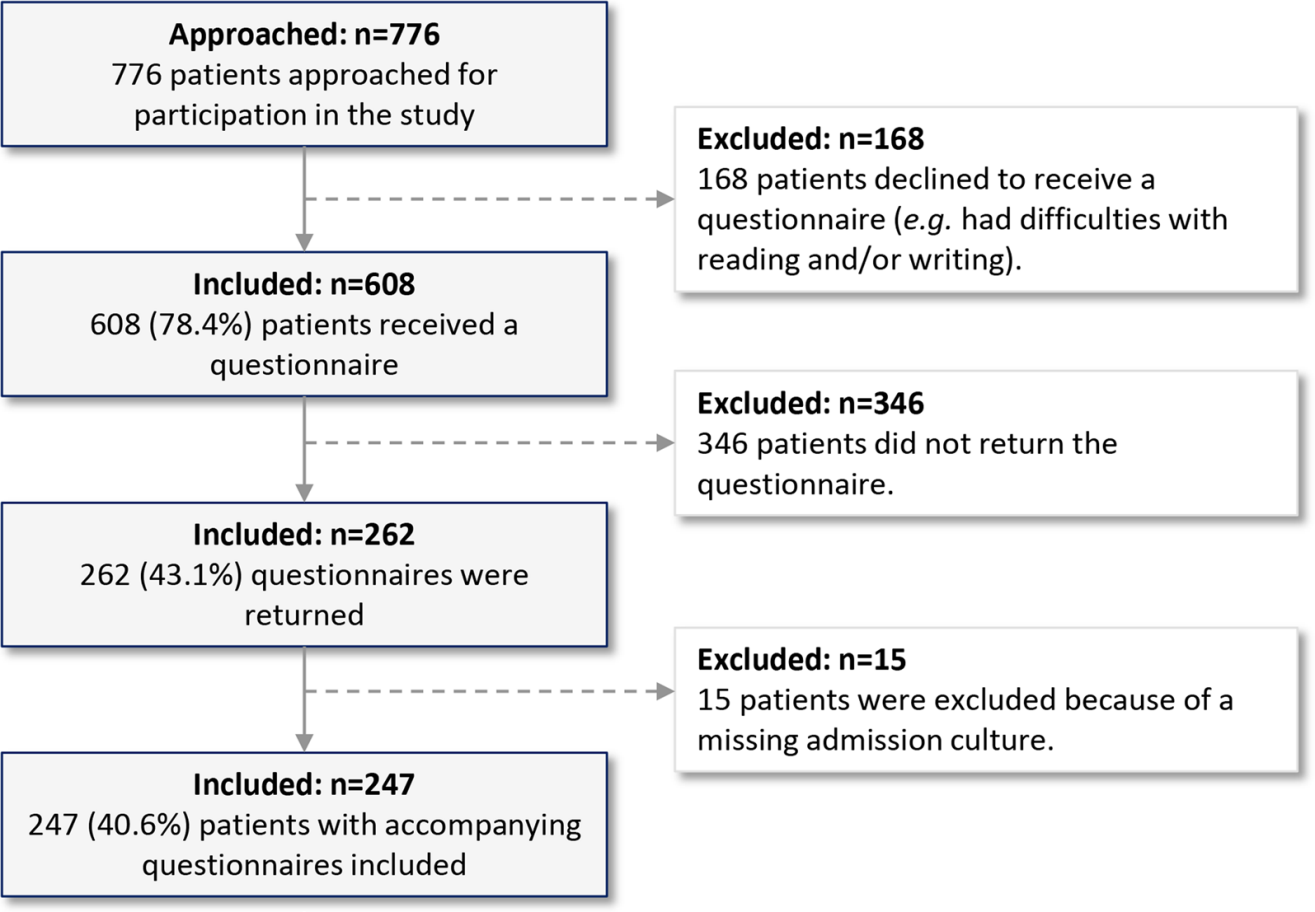
Flow diagram of patient inclusion

From May 18, 2018 until August 1, 2019, 776 patients were approached for participation, of which 608 (78.4%) received a travel questionnaire (Fig. 1). Out of 608 handed out questionnaires, 262 were returned (43.1%). In 27 out of 262 returned questionnaires (10.3%), one or more answers were missing. Fifteen questionnaires from 15 patients (5.7%) were excluded because of a missing admission culture. Therefore, 247 patients with accompanying questionnaires (247 out of 608, 40.6%) were included in the current study (Fig. 1).

Of the included patients, 141 were male (57.1%), and the median age of all included patients was 64 years (Table 1). One hundred twenty-two patients (52.6%) used antibiotics in the last year, and 106 patients (44.2%) used antacids in the last year (Table 1). Overall, fifteen out of 247 (6.1%) patients were HRMO carrier at admission; *n* = 12 (80%) carried ESBL-producing *E. coli*, *n* = 2 (13.3%) carried ESBL-producing *K. pneumoniae*, and *n* = 1 carried ESBL-producing *Proteus vulgaris* (6.7%). No other HRMO were detected. No significant differences were identified between characteristics of HRMO and non-HRMO carriers, including travelling abroad < 1 year before admission (*p*-value 0.995) (Table 1).

**Table 1:** Characteristics of patients carrying HRMO and patients not carrying HRMO at admission

Patient characteristic	HRMO carrier; n = 15	Not carrying HRMO; n = 232	*p*-value
Male gender (%)	10 (66.7)	131 (56.5)	0.439
Age, median (IQR)	64 (26)	64 (18)	0.273
Travel < 1y before admission (%)	7 (46.7)	110 (47.4)	0.955
Antibiotic use < 1y (%)	7^a^ (50)	115^b^ (52.5)	0.855
Antacid use < 1y (%)	9 (60)	97^c^ (42.9)	0.197
Travelling household members < 1y (%)	3 (20)	50 (21.6)	0.881
Animal contact^d^ (%)	2 (13.3)	86 (37.1)	NA
Domestic animal contact	2 (13.3)	72^e^ (31.4)	NA
Farm animal contact	0 (0)	5^e^ (2.2)	NA

NA, not applicable; y, year; HRMO, highly resistant microorganism; IQR, interquartile range

^a^One patient with missing information

^b^13 patients with missing information

^c^Six patients with missing information

^d^Contact with farm or domestic animals more than 3 times a week, more than 1 h each day

^e^Three patients with missing information; these patients only stated they had animal contact, but not with which animal

### Non-travelling patients

Hundred-and-thirty (52.6%) patients did not travel in the year before admission. Eight (6.2%) of these patients were HRMO carrier at admission; *n* = 5 carried an ESBL-producing *E. coli*, *n* = 2 carried an ESBL-producing *K. pneumoniae*, and *n* = 1 carried an ESBL-producing *P. vulgaris*. Non-travelling patients had significantly fewer household members that also travelled compared to patients that did travel (*p*-value 0.005, Table 2). Furthermore, non-travelling patients were significantly older compared to travelling patients (Table 2, Additional file 2).

**Table 2:** Patient characteristics of travelling and non-travelling patients

Patient characteristic	Travelling patient; n = 117	Non-travelling patient; n = 130	*p*-value
Male gender (%)	70 (59.8)	71 (54.6)	0.408
Age, median (IQR)	63 (21)	65 (15)	**0.006**
HRMO carrier at admission (%)	7 (6.0)	8 (6.2)	0.955
Antibiotic use < 1y (%)	57 (51.8)^a^	65 (52.8)^a^	0.875
Antacid use < 1y (%)	49 (43.0)^b^	57 (44.9)^b^	0.767
Travelling household members < 1y (%)	34 (29.3)^c^	19 (14.6)	**0.005**
Animal contact (%)^d,e^	46 (39.3)	42 (32.3)	0.251
Domestic animal contact	39 (33.9)^f^	35 (27.1)^g,h^	0.272
Dogs	26 (22.6)	24 (18.8)	0.463
Cats	21 (18.3)	21 (16.4)	0.708
Birds	2 (1.7)	3 (2.3)	NA
Rabbits	1 (0.9)	1 (0.8)	NA
Farm animal contact	2 (1.7)^g^	3 (2.3)^h^	NA
Horses	2 (1.7)	2 (1.6)	NA
Goats	1 (0.9)	1 (0.8)	NA
Poultry	1 (0.9)	1 (0.8)	NA
Sheep	0 (0)	2 (1.6)	NA
Pigs	0 (0)	1 (0.8)	NA

Significant differences are indicated in bold text

NA, not applicable; HRMO, highly resistant microorganism; y, year

^a^Seven patients with missing information

^b^Three patients with missing information

^c^One patient with missing information

^f^Contact with farm or domestic animals more than 3 times a week, more than 1 h each day

^e^Numbers do not add up because 20 patients had contact with multiple animals

^f^Two patients with missing information about which animal

^g^One patient with missing information about which animal

^h^One patient reported domestic animal contact but missing information about which animal

### Travelling patients

**Fig. 2:**
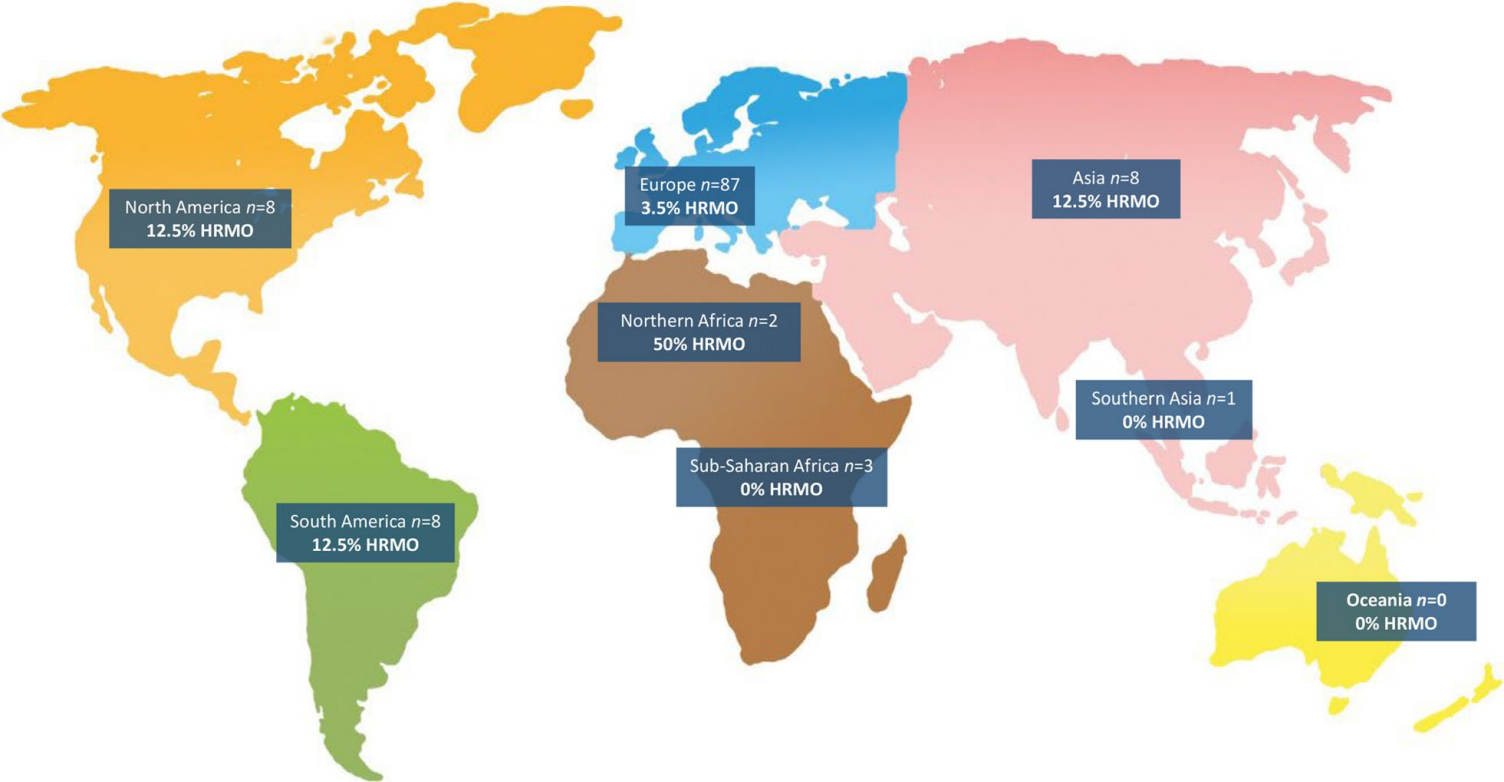
Regions visited by patients admitted to the Erasmus MC University Medical Center. HRMO; highly resistant microorganism

Out of the 247 patients, 117 patients (47.4%) travelled in the year before admission. Out of these 117 travelers, most patients (n = 87, 74.4%), travelled within Europe, and 30 patients (25.6%) travelled outside of Europe (Fig. 2). Of the 117 travelling patients, 54 patients (46.2%) travelled to multiple countries. Of these 54 patients, 38 patients (70.1%) travelled only within Europe, 15 patients (27.8%) travelled outside and inside Europe, and 1 patient (1.9%) travelled to multiple destinations outside of Europe. Most patients (n = 105 out of 117, 89.7%) travelled for less than 1 month (Table 3).

**Table 3:** Travel behavior of travelling patients carrying HRMO at admission compared to not carrying HRMO at admission

Characteristic	Total n = 117	HRMO-positive at admission, n = 7	HRMO-negative at admission, n = 110
**Duration T < 1 month**	**105 (89.7)**	7 (100)*	98 (89.1)*
Duration T 1–3 months	9 (7.7)	0 (0)	9 (8.2)
Duration T 3–6 months	2 (1.7)	0 (0)	2 (1.8)
Duration T 6–12 months	1 (0.9)	0 (0)	1 (0.9)
**Travelling outside of Europe < 1y**	**30 (25.6)**	**4 (57.1)**	26 (23.6)**
**Travelling within Europe < 1y**	**87 (74.4)**	**3 (42.9)**	**84 (76.4)**
Ice cream and pastry consumption (%)	64^c^ (56.1)	3^a^ (50)	61^b^ (56.5)
Meals at street food stalls (%)	10^a^ (8.6)	0 (0)	10^a^ (9.2)
Experienced vomiting during travel (%)	3^a^ (2.6)	0 (0)	3^a^ (2.8)
Experienced diarrhea during travel (%)	6^a^ (5.2)	1 (14.3)	5^a^ (4.6)
Admitted to hospital during travel (%)	8 (6.8)	0 (0)	8 (7.3)
Antibiotic use during travel (%)	6^b^ (5.2)	1 (14.3)	5^b^ (4.6)
Antacid use during travel (%)	22^c^ (19.3)	1^a^ (16.7)	21^b^ (19.4)
Used malaria prophylaxis during travel (%)	1^a^ (0.9)	0 (0)	1^a^ (0.9)

Relevant differences in percentages indicated in bold text

Duration T, duration of travel; HRMO, highly resistant microorganism; y, year

^a^One patient answered this question with ‘unknown’

^b^Two patients answered this question with ‘unknown’

^c^Three patients answered this question with ‘unknown’

*P-value 0.356. **Chi-square *P*-value 0.049, Fisher’s exact test *P*-value 0.070

In total, seven out of 117 travelling patients (6.0%) were HRMO carrier at hospital admission. All seven travelling patients carried an ESBL-producing *E. coli*, and travelled for less than a month (Table 3). Thirty out of 117 patients (25.6%) travelled outside of Europe; in this group the HRMO prevalence was 13.3% (4 out of 30; all ESBL-positive *E. coli*). The highest carriage rates were observed in patients travelling to Northern Africa (50%), followed by travelling to Asia, to North America, and to South America 12.5%) (Fig. 2), but overall carriage rates were low.

Out of 117 patients, 107 patients (91.5%) replied that the questionnaire was clear and easy, and 10 patients (8.5%) replied that they had difficulties to recall all the asked information (mainly the questions about use of antibiotics and antacids).

### Behavior during travel

Overall, more than half of the travelling patients consumed ice cream and/or pastries during travel (Table 3). Travelling patients carrying HRMO at hospital admission experienced, with low numbers of patients however, more often diarrhea (14.3% vs. 4.6%), and used more often antibiotics during travel (14.3% vs. 4.6%) compared to patients not carrying HRMO at admission (Table 3). Vomiting during travel and the use of malaria prophylaxis were only described in HRMO-negative patients. Additionally, only HRMO-negative patients reported that they ate meals at street food stalls. HRMO carriage rates were higher for patients travelling outside of Europe, compared to patients travelling in Europe (13.3% vs. 3.4%, Table 3).

### Genomic analysis

WGS results confirmed the presence of beta-lactamases in the isolates from the 12 patients identified with an ESBL-producing *E. coli* (Additional file 3: Fig. 1, Additional file 4: Fig. 2). The beta-lactamases distribution in isolates was not associated with patient travelling (Additional file 3: Fig. 1). In two travelling and one non-travelling patient (patients 1, 2 and 3) *bla*_OXA-1_ was detected. These three isolates also contained an *bla*_CTX-M-15_ and *aac(6’)-Ib-cr* gene. Additionally, multiple other aminoglycoside-modifying enzymes (AMEs) were present in these 12 isolates with their presence being independent of travelling (Additional file 4: Fig. 2). We observed that isolates of patients 6 and 7 did not possess any AME, and the isolate of patient 8 that had only one AME (ANT(3″)-IIa). The isolates of these three patients were of the same sequence type (ST)69. Other antimicrobial genes identified were *ampC*, *tet(A)*, *tet(B)*, and *tetR*, which were present in isolates from travelling and non-travelling patients. The isolates from one travelling patient (patient 4) and one non-travelling patient (patient 11) lacked these additional antimicrobial resistance genes (Additional file 4: Fig. 2). Two ESBL-producing *K. pneumoniae* isolates were found in non-travelling patients. One isolate belonged to ST465, and contained *bla*_TEM-1_ *bla*_CTX-M-15_ and *bla*_SHV-1_, and the other isolate belonged to ST1565 and contained *bla*_OXA-1_, *bla*_DHA-1_ and *bla*_SHV-64_. For the ESBL-producing *P. vulgaris* no known ESBL genes were detected using the CARD database v3.0.5. However, using the disk diffusion ESBL kit (Rosco Diagnostica, Taastrup, Denmark), ESBL production was confirmed phenotypically.

### Risk perception

The majority of patients (n = 176 out of 247; 71.3%) were aware that international travel could lead to carriage of HRMO. The majority of patients (221 out of 243; 90.9%) supported the idea to screen for HRMO upon hospital admission in case of a travel history; 4 patients (1.6%) did not answer this question.

Travelling HRMO positive patients were less aware of the fact that travelling could lead to HRMO carriage (57.1% compared to 68.2%). Additionally, they were more careless with respect to perception of risk (Table 4). In both groups, approximately 86% supported the idea to screen for HRMO upon hospital admission in case of a travel history (Table 4).

**Table 4:** Risk perception of travelling patients in relation to HRMO positivity at admission

Opinion about risk of acquiring HRMO after travel	HRMO-positive at admission, n = 7	HRMO-negative at admission, n = 110
Aware that travel could lead to HRMO acquisition (%)	**4 (57.1)**	**75 (68.2)**
Risk of acquiring HRMO is no problem (%)	0 (0)	5 (4.5)
Aware that travel comes with risks (%)	**3 (42.9)**	**31 (28.2)**
Unpleasant, but will still travel (%)	**1 (14.3)**	**57 (51.8)**
Risk of acquiring HRMO is scary (%)	1 (14.3)	7 (6.4)
Other, or combination of answers (%)	2 (28.6)	10 (9.1)
Hospitals should screen for HRMO in case of a travel history (%)	6 (85.7)	94^a^ (86.2)

Relevant differences in percentages indicated in bold text

HRMO; highly resistant microorganism

^a^One missing answer

## Discussion

### Summary of evidence

Our study showed that almost 50% of the patients admitted to the hospital travel, both within and outside of Europe. Overall, we did not show a difference in carriage rates at admission between travelling < 1y to any country abroad and non-travelling patients. Multiple studies have determined the effect of travel on ESBL acquisition, and highlighted the importance of improved screening and efforts to reduce import [4]. However, information on acquisition of HRMO during travel of patients is scarce; even more because other studies focused on people in settings outside hospitals, such as travel clinics. We found an overall carriage rate of 6.1%; 6.2% for non-travelers and 6.0% for travelling patients, which is comparable to the normal carriage rate of ESBL-producing Enterobacterales in the Netherlands [8]. However, the majority of patients travelled within Europe. While the prevalence of HRMO is higher in Southern European countries compared to the Netherlands and countries in the Northern part of Europe, research has shown that travelling to countries in especially South East Asia is a risk factor [4]. We showed that patients that did travel outside of Europe had higher carriage rates upon admission, compared to patients travelling in Europe, and compared to patients that did not travel (13.3% vs. 3.4% vs. 6.2%).

With regard to patients that did travel, experiencing diarrhea or vomiting during travel were rare, as was being admitted to a hospital abroad (*i.e.* less than 7%). Out of six patients using antibiotics abroad, only one carried an HRMO upon admittance. This in contrast to the study by Wuerz et al*.* that described that the risk of acquiring ESBL-producing Enterobacterales increases substantially when using antibiotics during travel [9]. Overall, more than 50% of patients used antibiotics in the year before admission. This could be considered as high, especially higher compared to the study by Reuland et al., who took a representative sample of the general adult Dutch population and found rates between 14 and 26% [10]. The difference between our findings and the findings by Reuland et al*.* could be explained by different populations included; in our study this population included patients of a tertiary care hospital. Additionally, the median age of included patients was 64 years old, ranging from 20 to 91, which is considerably older compared to the study by Reuland et al*.* (i.e. median age of cases 48 and controls 50 years old) and by Arcilla et al., (i.e. 51 years old, range 33 to 61). We assume that our older, tertiary-care hospital patients were less likely travelers outside of Europe.

We identified that two out of seven travelers carrying an ESBL-producing *E. coli* carried *E. coli* ST131, a common strain in the world, including in the Dutch community, and no carbapenemase-producing isolates were identified. In the study by Arcilla et al*.*, and Peirano et al*.*, *bla*_CTX-M-15_ was the most frequently acquired ESBL-gene in travelers (> 50%), as was in our study (6 out of 12 ESBL-producing *E. coli*, 50%; 4 travelling patients and 2 in non-travelling patients) [3, 11]. CTX-M-15 (CTX-M-1 group) and CTX-M-27 (CTX-M-9 group) were previously identified as prevalent in the Netherlands, including in long-term care facilities, while CTX-M-14/65 (CTX-M-9 group and CTX-M-55 (CTX-M-1 group) are less present in the Dutch population [10, 12–14]. In three patients, *bla*_OXA-1_ was found, in combination with *bla*_CTX-M-15_ and *aac(6’)-Ib-cr,* which was also described as being a frequent combination in the UK [15]. Of these, the *aac(6’)-Ib-cr* is most worrisome, as this enzyme also confers resistance to ciprofloxacin and norfloxacin and its gene is known to be plasmid-mediated.

### Towards a guideline: part 2

In a previous study, we described knowledge gaps that needed to be filled before national and international guidelines could be developed [4]. First, we described that the proportion of patients with a recent travel history is unknown. With this current study, we identified that almost 50% of admitted patients travelled abroad in the last year, of which 25.6% travelled outside of Europe. Second, we previously described that it is unknown if strains carried by travelers spread in hospitals. In this study, we did not include ward mates nor did we sample the environment to assess spread in the hospital, so this knowledge gap is still unfilled. Third, the threshold of a carriage rate after travel that warrants screening and/or isolation was also an unresolved issue. In this study, we showed that carriage rates were higher in patients that travelled to Northern Africa, Asia, North America, and to South America in the last year, than the ESBL carriage rate in the Dutch community (*i.e.* 5.3%-9.9%) [8]. In a study prospectively including healthy travelers, ESBL carriage rates observed among people travelling to Southeastern Asia (31.6%), followed by Southern Asia (21.5%), were higher than in the Dutch community [3]. This could point to a strategy of only preemptively screening and isolating patients that have travelled to those countries.

A high majority of patients support the idea to screen for HRMO upon hospital admission in case of a travel history. However, although patients support screening, it is questionable if preemptive isolation and screening for around 12% (*i.e.* 30 out of 247 patients) of all admitted patients because of travelling outside of Europe in the last year is cost-effective, and even feasible in many hospitals with respect to isolation capacity. A screening-only (*i.e.* without preemptive isolation) policy could be considered, with as draw back that a contact investigation must be performed when an HRMO-positive patient is identified. We chose to ask for travelling in the year before hospital admission, however, also different cut-offs can be used (*e.g.* 1 month, 2 months, 3 months), since literature shows that the median elimination time of HRMO carriage after travel is quick [16]. Therefore, we calculated the percentages of HRMO carriage when selecting more focused target populations for screening, primarily focusing on travelling to Asia or Africa, as previously defined destinations with high HRMO carriage upon return [3]. Percentages of HRMO carriage increased when travel was closer to hospital admission, for patients travelling outside Europe and for patients travelling to Asia or Africa (*i.e.* travel outside Europe: 13% [n = 4/30] if travelled < 1 year before hospital admission to 29% [n = 2/7] if < 3 months to 40% [n = 2/5] if < 2 months to 67% [n = 2/3] if < 1 month; Travel to Asia or Africa: 14% [n = 2/14] if travelled < 1 year before hospital admission to 33% [n = 1/3] if < 3 months to 50% [n = 1/2] if < 2 months to 100% [n = 1/1] if < 1 month). Additionally, the numbers of patients included in these groups decrease rapidly. Antibiotic use during the year before hospital admission was not related to HRMO carriage. Considering the results of this current study and discussed literature, we would propose to target the patients that travelled more recently (*i.e.* < 2 months) for screening and preemptive isolation. The travel destinations to include could be any country outside Europe based on our limited data, or travel to Asia or Africa, based on the broader picture from published data in combination with our data. A strategy with a more targeted patient population will be feasible for many hospitals, and most likely be cost-effective.

### Strengths and limitations

A strength of our study is that we included a reasonable large number of patients with information on travel history with an accompanying admission culture. However, since we did not sample the patients before and after travel but at hospital admission, we do not know whether patients were already carrying an HRMO, or acquired the HRMO during travel. A second strength is that we asked for the perception of the patients towards this subject.

Potential limitations include this being a single center study in a tertiary care hospital, including a relatively older patient population with complicated medical histories who might travel less often compared to patients admitted to secondary care hospitals. Second, only a low number of HRMO were identified. This could mean that this study was underpowered and could therefore not identify meaningful differences between groups Therefore, the results of this study should be confirmed by a larger study. Third, we could have encountered recall bias of patients with regard to questionnaire, and finally, we have introduced a language bias by providing the questionnaire in Dutch only.

## Conclusions

With this study, we identified that half of admitted patients to a large tertiary care hospital travelled abroad in the last year, with only a small percentage outside Europe. We discussed that a strategy including screening and preemptive isolation of patients who travelled to Asia or Africa in the previous 2 months could be considered. Also, we learned that this strategy would be supported by patients. Some previously identified knowledge gaps have been filled and we are one step closer towards a guideline. However, before national or international guidelines can be developed, future research should focus on determining the burden of disease of travel-related HRMO carriage, and its transmissibility to other patients and to the environment, using a multi-center study design and taking cost-effectiveness into account. Finally, since this study was performed before the COVID-19 pandemic it is unknown if travel behavior changed because of this, and if travel destinations changed. Therefore, post-COVID studies still have to be performed, to assess the impact of the COVID-19 pandemic.

## Supplementary Information

**Additional file 1**. Patient information form and questionnaire (in Dutch).

**Additional file 2**. Age distribution between travelling patients and non-travelling patients.

**Additional file 3**. Minimum spanning tree representing cgMLST analysis of the ESBL-producing *E. coli* strains. Node numbers correspond to patient numbers and line numbers indicate the number of different alleles between strains. Colors match the sequence types (ST). A grey background indicates genetically closely related isolates.

**Additional file 4**. Distribution of selected antimicrobial resistance genes among the *E. coli* isolates. Isolates from patients are clustered based on similarities of presence and absence of the antimicrobial resistance genes. Blue represents a perfect hit to the reference sequence in the CARD database, teal represents a strict hit, and blank indicates absence of that gene in the isolate (8). Patient 9 was included twice in the study. ESBL-positive *E. coli* were cultured on both admissions (9a and 9b).

## Data Availability

All data generated or analyzed during this study are included in this published article and its additional files, or can be provided by the corresponding author upon reasonable request.

## Abbreviations

AMEs: Aminoglycoside-modifying enzymes
CIM: Carbapenem inactivation method
Erasmus MC: Erasmus MC University Medical Center, Rotterdam, The Netherlands
ESBL: Extended-spectrum beta-lactamase
HRMO: Highly resistant microorganisms
ST: Sequence type
WGS: Whole genome sequencing
