# Supplementary material for: Pre-COVID-19 international travel and admission to hospital when back home: travel behavior, carriage of highly resistant microorganisms, and risk perception of patients admitted to a large tertiary care hospital
Source: Antimicrob Resist Infect Control. 2022 Jun 2;11:78. doi: 10.1186/s13756-022-01106-x (PMC9161189; doi:10.1186/s13756-022-01106-x)
Supplement: Supplementary file 1 — Additional file 1. Patient information form and questionnaire (in Dutch). [file 13756_2022_1106_MOESM1_ESM.pdf]

Betreft: Informatie over bijgevoegde vragenlijst:

Reisgedrag van patiënten 12 maanden voor ziekenhuisopname en risicofactoren voor dragerschap van een resistente bacterie.

Geachte heer/mevrouw,

Wij vragen u vriendelijk om mee te doen aan een medisch-wetenschappelijk onderzoek, ook wel een studie genoemd. U beslist zelf of u wilt meedoen. Maar voordat u de beslissing neemt, is het belangrijk om meer te weten over het onderzoek. Neem de tijd om deze informatie te lezen, te bespreken met anderen en aarzel niet om vragen te stellen. Onderaan deze brief vindt u onze contactgegevens.

1. Wat is het doel van dit onderzoek?

Mensen die reizen buiten Nederland maken nemen soms bacteriën in hun darmen mee terug die ongevoelig (resistent) zijn voor de meest gebruikte antibiotica. Dit is in meerdere onderzoeken gevonden. Van deze bacteriën krijgen mensen meestal geen klachten. Bij opname in een ziekenhuis is de kans aanwezig dat zij deze resistente bacteriën overdragen aan hun omgeving of andere patiënten. In Nederland is voor deze patiënten geen landelijk beleid bij opname in een ziekenhuis, omdat er nog veel onduidelijk is. Zo weten we weinig over het reisgedrag van patiënten. In dit onderzoek willen wij het reisgedrag in kaart brengen van patiënten die in het Erasmus MC worden opgenomen. We richten ons op het reisgedrag van de afgelopen 12 maanden. We stellen ook vragen over gebruik van medicijnen en diercontact, omdat dit ook een invloed kan hebben op de darmflora.

2. Wat wordt er van u verwacht?

Tijdens uw opname wordt u gevraagd een vragenlijst in te vullen, deze ingevuld in de bijgeleverde envelop te stoppen en deze daarna kosteloos op te sturen. Dit kan ook na uw opname.

3. Wat zijn mogelijke voor- en nadelen van deelname aan dit onderzoek?

U heeft zelf geen voordeel van deelname aan dit onderzoek. Voor de toekomst kan het onderzoek wel nuttige gegevens opleveren. Een nadeel van deelname is de tijd die u nodig heeft voor het invullen van de vragenlijst. Uw behandeling zal niet veranderen door de antwoorden die u geeft op de vragen; alleen het onderzoeksteam verwerkt uw antwoorden, uw behandelend arts kan deze vragenlijst niet inzien. Ook worden uw gegevens anoniem verwerkt.

4. Wat gebeurt er als u niet wenst deel te nemen aan dit onderzoek?

U beslist zelf of u meedoet aan het onderzoek. Deelname is vrijwillig. Als u besluit niet mee te doen, hoeft u verder niets te doen. U hoeft ook niet te zeggen waarom u niet wilt meedoen. Als u wel meedoet, kunt u zich altijd bedenken en toch stoppen. Ook tijdens het onderzoek.

5. Wat gebeurt er met uw gegevens?

Uw onderzoeksgegevens kunnen alleen door bevoegde personen worden ingezien. Dit zijn medewerkers van het onderzoeksteam, medewerkers van de Inspectie Gezondheidszorg en Jeugd (IGJ) en leden van de Medisch Ethische Toetsingscommissie (METC) van het Erasmus MC. Deze inzage is mogelijk nodig om de betrouwbaarheid en kwaliteit van het onderzoek na te gaan. De onderzoeksgegevens zullen worden gehanteerd met inachtneming van de Wet Bescherming Persoonsgegevens en het privacyreglement van het Erasmus MC. Persoonsgegevens die tijdens deze studie worden verzameld, zullen door een codenummer worden vervangen. Alleen het codenummer wordt gebruikt voor studiedocumentatie, rapporten, of publicaties over dit onderzoek. De sleutel van deze codenummers is alleen toegankelijk voor de onderzoekers. De vertrouwelijkheid van de gegevens blijft hierbij gewaarborgd.

6. Zijn er extra kosten of is er een vergoeding wanneer u besluit aan dit onderzoek mee te doen?

Aan deelname aan dit onderzoek zijn geen kosten van uw kant verbonden. Er is geen vergoeding voor deelname.

7. Welke medisch-ethische toetsingscommissie heeft dit onderzoek goedgekeurd?

De Medisch Ethische Toetsingscommissie (METC) van het Erasmus MC heeft voor dit onderzoek een verklaring 'niet WMO-plichtig onderzoek' afgegeven. Dat betekent dat dit onderzoek door de onderzoeker is aangemeld bij deze METC en niet valt onder de wet medisch-wetenschappelijk onderzoek met mensen.

8. Wilt u verder nog iets weten?

Mocht u vragen of opmerkingen hebben, aarzel dan niet deze te stellen. U kunt tijdens kantooruren contact opnemen met uitvoerend onderzoeker Dr. Anne Voor in 't holt, afdeling Medische Microbiologie en Infectieziekten, via e-mail a.voorintholt@erasmusmc.nl. U kunt uw vragen eventueel ook stellen aan Dr. Juliëtte Severin, afdeling Medische Microbiologie en Infectieziekten, via e-mail: j.severin@erasmusmc.nl of telefonisch: 010-7033510.

Indien u na zorgvuldige overweging besluit deel te nemen aan dit wetenschappelijk onderzoek, dan vragen we u om de vragenlijst in te vullen en op te sturen met behulp van de meegeleverde antwoordenvelop.

Met vriendelijke groet, het onderzoeksteam

Naam:………………………………………………………………………………………

Geboortedatum:………………………………………………Man/Vrouw

Datum invullen vragenlijst: ……………………-…………………-2019

In de darmen van mensen komen veel bacteriën voor, dit wordt de darmflora genoemd. Bij reizen naar het buitenland kan de darmflora veranderen. Bacteriën die ongevoelig (resistent) zijn voor antibiotica komen in het buitenland meer voor dan in Nederland. Deze resistente bacteriën kunnen dan onderdeel worden van de darmflora, zonder dat je daar iets van merkt.

1) Wist u dat bij reizen naar het buitenland mensen ongemerkt drager kunnen worden van een resistente bacterie in de darmen?

☐ Ja

☐ Nee

2) Wat vindt u daarvan?

☐ Geen probleem

☐ Reizen heeft nu eenmaal risico's

☐ Geen prettig idee, maar ik vind reizen leuk/belangrijk

☐ Eng

☐ Anders, namelijk: …………………………………………………

3) Vindt u dat ziekenhuizen in Nederland patiënten hierop moeten controleren? *(Bijvoorbeeld vragen naar reizen en/of kweken van bacteriën uit de darm bij opname)*

☐ Ja

☐ Nee

4) Heeft u meer dan 3x per week, meer dan 1 uur per dag contact met dieren? *(Bijvoorbeeld honden, katten, varkens, koeien, pluimvee)*

☐ Ja, namelijk met…………………………………………………

☐ Nee

5) Heeft u in de laatste **12 maanden** antibiotica gebruikt? *(Zie vraag 15 voor voorbeelden)*

☐ Ja

☐ Nee

☐ Weet ik niet

6) Heeft u in de laatste **12 maanden** maagzuurremmers gebruikt? *(Bijvoorbeeld omeprazol, Losec, Nexium*)

☐ Ja

☐ Nee

☐ Weet ik niet

7) Heeft u huisgenoten die de laatste **12 maanden** zonder u naar het buitenland hebben gereisd?

☐ Ja

☐ Nee

8) Heeft u de afgelopen **12 maanden** naar het buitenland gereisd?

☐ Ja → **Ga door naar vraag 9**

☐ Nee → **Einde vragenlijst, hartelijk dank voor uw medewerking!**

9) Hoeveel vakanties/reizen heeft u de afgelopen 12 maanden gemaakt, welk(e) land(en) heeft u bezocht en wat was de vertrek- en terugkomstdatum?

- Heeft u meerdere landen bezocht gedurende 1 vakantie/reis, dan geldt dit als 1 vakantie/reis.
- Als u niet meer de exacte vertrek en terugkomstdatum weet, mag u ook het aantal dagen opschrijven.

**Nr.**	**Land(en)**	**Vertrekdatum**	**Terugkomstdatum**	**Soort reis***
**1**				
**2**				
**3**				
**4**				
**5**				

**Soort reis: bijvoorbeeld backpacken, groepsreis, strandvakantie, stedentrip, zakenreis, bezoek familie/vrienden, all-inclusive reis.*

10) Heeft u gebak of onverpakt ijs gegeten **tijdens uw vakantie/reis**?
☐ Ja, bij vakantie/reis nummer(s)………..
☐ Nee
☐ Weet ik niet

11) Heeft u **tijdens uw vakantie/reis** eten van straatstalletjes gegeten?
☐ Ja, bij vakantie/reis nummer(s)………..
☐ Nee
☐ Weet ik niet

12) Heeft u **tijdens uw vakantie/reis** last gehad van braken/overgeven?
☐ Ja, bij vakantie/reis nummer(s)………..
☐ Nee
☐ Weet ik niet

13) Heeft u **tijdens uw vakantie/reis** last gehad van diarree?
☐ Ja, bij vakantie/reis nummer(s)………..
☐ Nee
☐ Weet ik niet

14) Bent u in een ziekenhuis opgenomen geweest **tijdens uw vakantie/reis**?
☐ Ja, bij vakantie/reis nummer(s)……….., aantal dagen opgenomen geweest………….
☐ Nee
☐ Weet ik niet

15) Heeft u antibiotica gebruikt **tijdens uw vakantie/reis**?

☐ Ja → vul onderstaande tabel in

☐ Nee → ga door naar vraag **16**

☐ Weet ik niet → ga door naar vraag **16**

**Vakantie/reis nummer(s)** ***zie vraag 9***	**Naam antibioticum***	**Reden****	**Toediening*****	**Hoe gekregen?******

* *Bijvoorbeeld: Amoxicilline, Amoxicilline/clavulaanzuur (Augmentin), Ciprofloxacine (Ciproxin), Nitrofurantoïne (Furabid), Doxycycline, Claritromycine (Klacid), Azitromycine (Zithromax), Trimethoprim, Cotrimoxazol (Bactrimel).*

** *Bijvoorbeeld: Diarree, longontsteking, verkoudheid, huidinfectie, koorts.*

*** *Bijvoorbeeld: Tabletten, infuus, zalf, drankje.*

**** *Bijvoorbeeld: Voorgeschreven door arts in het buitenland, via een apotheek (zonder recept), via een drogist, van huis meegenomen naar het buitenland.*

16) Heeft u maagzuurremmers *(bijvoorbeeld omeprazol, Losec, Nexium*) gebruikt **tijdens uw vakantie/reis**? Dan wel van huis meegenomen, voorgeschreven gekregen in het buitenland of gekocht tijdens uw vakantie/reis.

☐ Ja, bij vakantie/reis nummer(s)………..

☐ Nee

☐ Weet ik niet

17) Heeft u malariaprofylaxe gebruikt? (Bijvoorbeeld Malarone, Lariam, Doxycycline)

☐ Ja, bij vakantie/reis nummer(s)………..
Welke profylaxe heeft u gebruikt? …………………………………………………..

☐ Nee

☐ Weet ik niet

18) Vond u het lastig om antwoord te geven op de vragen in deze vragenlijst?

☐ Nee

☐ Ja, veel details, wist het niet meer precies

☐ Ja, vragen onduidelijk, met name vraag/vragen……………………………….

☐ Ja, omdat………………………………………………………………………………….

Einde vragenlijst. Hartelijk dank voor uw medewerking!
