# Supplementary material for: Pre-COVID-19 international travel and admission to hospital when back home: travel behavior, carriage of highly resistant microorganisms, and risk perception of patients admitted to a large tertiary care hospital
Source: Antimicrob Resist Infect Control. 2022 Jun 2;11:78. doi: 10.1186/s13756-022-01106-x (PMC9161189; doi:10.1186/s13756-022-01106-x)

**Additional file 2**. Age distribution between travelling patients and non-travelling patients.

| **Travel** | **Statistic** | **Value** |
| --- | --- | --- |
| No (0)  n=130 patients | Mean | 63.08 |
|  | 95% Confidence interval for mean lower bound | 60.88 |
|  | 95% Confidence interval for mean upper bound | 65.27 |
|  | Median | 65.00 |
|  | Standard deviation | 12.66 |
|  | Minimum | 22 |
|  | Maximum | 91 |
|  | Interquartile range | 15 |
| Yes (1)  n=117 patients | Mean | 57.87 |
|  | 95% Confidence interval for mean lower bound | 55.26 |
|  | 95% Confidence interval for mean upper bound | 60.49 |
|  | Median | 63.00 |
|  | Standard deviation | 14.28 |
|  | Minimum | 20 |
|  | Maximum | 82 |
|  | Interquartile range | 21 |


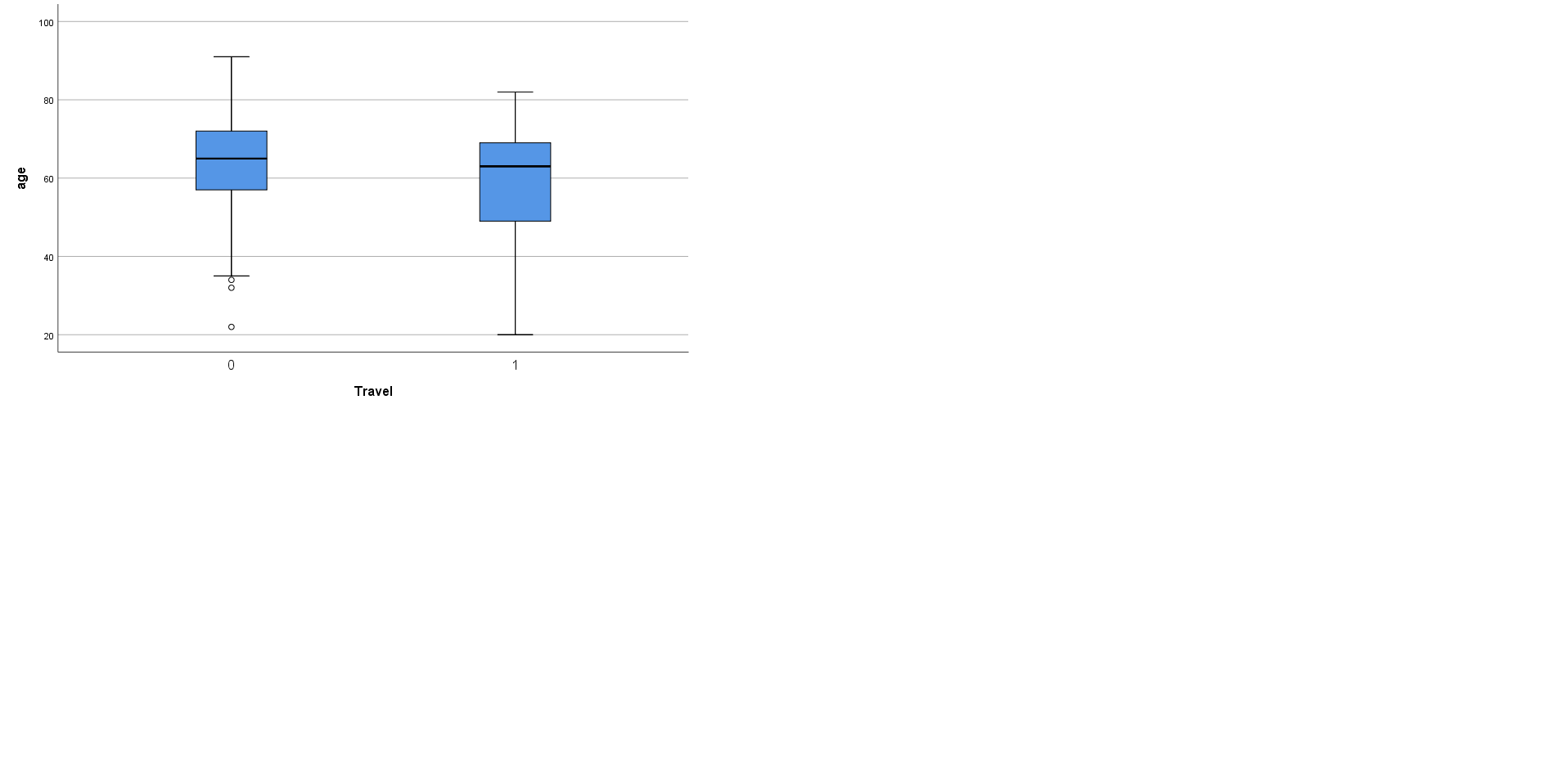

Supplement: Supplementary file 2 — Additional file 2. Age distribution between travelling patients and non-travelling patients. [file 13756_2022_1106_MOESM2_ESM.docx]
