# Supplementary figures and images for: Pre-COVID-19 international travel and admission to hospital when back home: travel behavior, carriage of highly resistant microorganisms, and risk perception of patients admitted to a large tertiary care hospital
Source: Antimicrob Resist Infect Control. 2022 Jun 2;11:78. doi: 10.1186/s13756-022-01106-x (PMC9161189; doi:10.1186/s13756-022-01106-x)

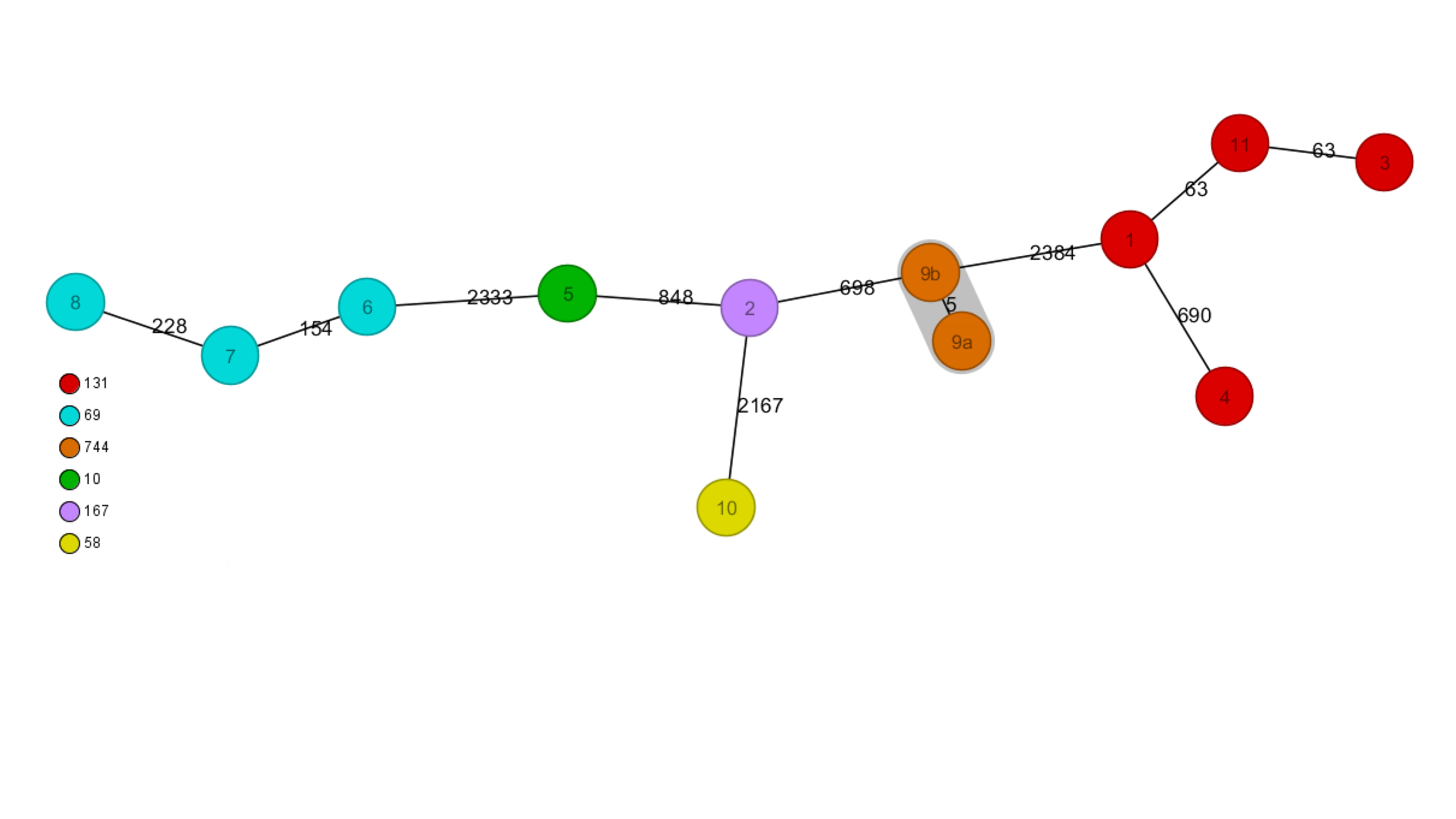

Supplement: Supplementary file 3 — Additional file 3. Minimum spanning tree representing cgMLST analysis of the ESBL-producing E. coli strains. Node numbers correspond to patient numbers and line numbers indicate the number of different alleles between strains. Colors match the sequence types (ST). A grey background indicates genetically closely related isolates. [file 13756_2022_1106_MOESM3_ESM.png]

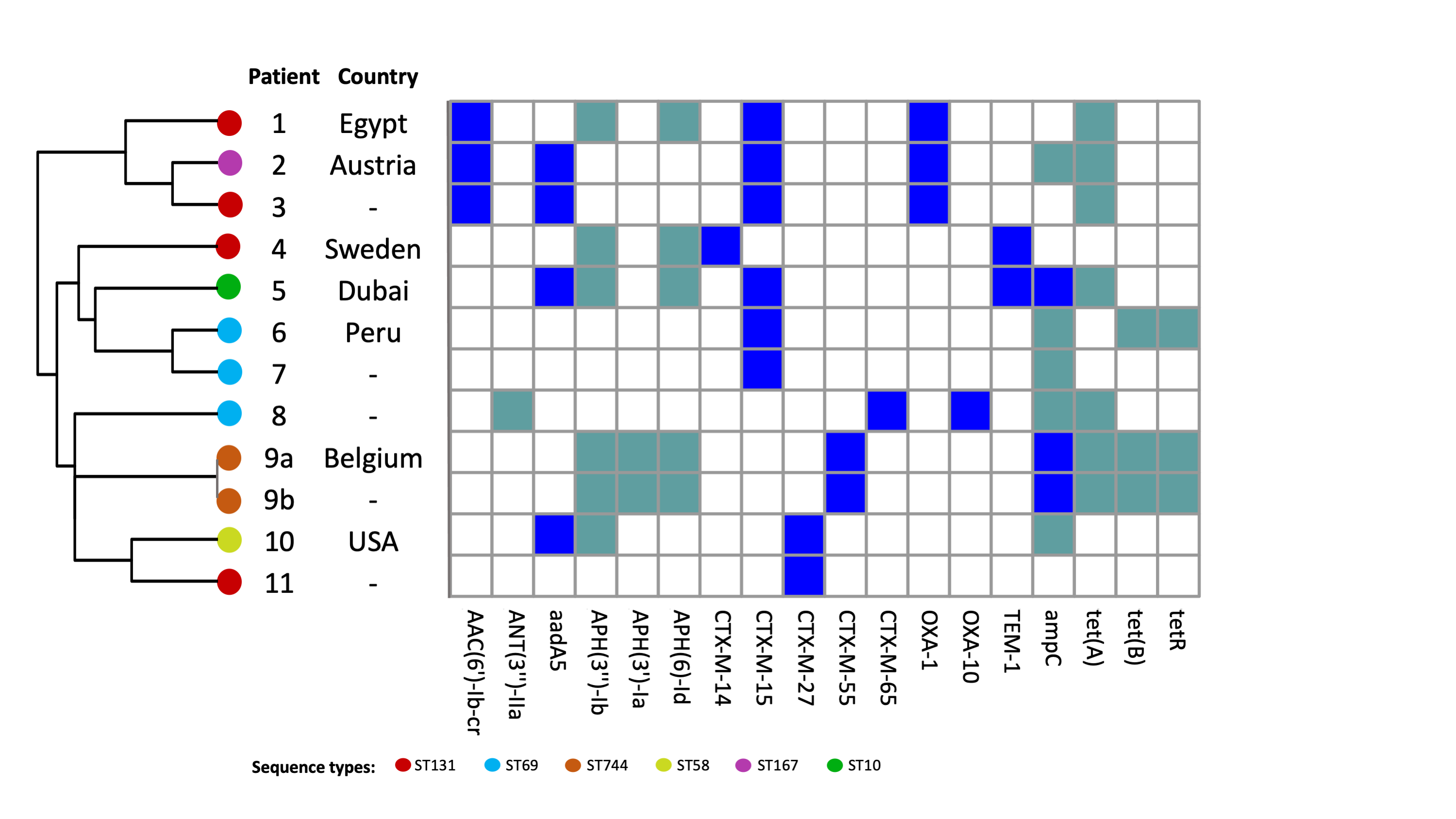

Supplement: Supplementary file 4 — Additional file 4. Distribution of selected antimicrobial resistance genes among the E. coli isolates. Isolates from patients are clustered based on similarities of presence and absence of the antimicrobial resistance genes. Blue represents a perfect hit to the reference sequence in the CARD database, teal represents a strict hit, and blank indicates absence of that gene in the isolate (8). Patient 9 was included twice in the study. ESBL-positive E. coli were cultured on both admissions (9a and 9b). [file 13756_2022_1106_MOESM4_ESM.tif]
